# Embryonic Stem Cell Differentiation to Definitive Endoderm As a Model of Heterogeneity Onset During Germ Layer Specification

**DOI:** 10.32607/actanaturae.27510

**Published:** 2024

**Authors:** M. N. Gordeev, A. S. Zinovyeva, E. E. Petrenko, E. V. Lomert, N. D. Aksenov, A. N. Tomilin, E. I. Bakhmet

**Affiliations:** Pluripotency Dynamics Group, Institute of Cytology, Russian Academy of Sciences, St. Petersburg, 194064 Russian Federation; Laboratory of the Molecular Biology of Stem Cells, Institute of Cytology, Russian Academy of Sciences, St. Petersburg, 194064 Russian Federation; Institute of Evolution, University of Haifa, Haifa, 3498838 Israel; Faculty of Biology, Technion – Israel Institute of Technology, Haifa, 3200003 Israel; Laboratory of Molecular Medicine, Institute of Cytology, Russian Academy of Sciences, St. Petersburg, 194064 Russian Federation; Department of Intracellular Signaling and Transport, Institute of Cytology, Russian Academy of Sciences, St. Petersburg, 194064 Russian Federation

**Keywords:** pluripotency, specification, differentiation, embryonic stem cells, ESCs, CRISPR/Cas9, gastrulation, endoderm, Foxa2

## Abstract

Embryonic stem cells (ESCs) hold great promise for regenerative medicine thanks to their ability to self-renew and differentiate into somatic cells and the germline. ESCs correspond to pluripotent epiblast — the tissue from which the following three germ layers originate during embryonic gastrulation: the ectoderm, mesoderm, and endoderm. Importantly, ESCs can be induced to differentiate toward various cell types by varying culture conditions, which can be exploited for *in vitro *modeling of developmental processes such as gastrulation. The classical model of gastrulation postulates that mesoderm and endoderm specification is made possible through the FGF-, BMP-, Wnt-, and Nodal-signaling gradients. Hence, it can be expected that one of these signals should direct ESC differentiation towards specific germ layers. However, ESC specification appears to be more complicated, and the same signal can be interpreted differently depending on the readout. In this research, using chemically defined culture conditions, homogeneous naïve ESCs as a starting cell population, and the *Foxa2 *gene-driven EGFP reporter tool, we established a robust model of definitive endoderm (DE) specification. This *in vitro *model features formative pluripotency as an intermediate state acquired by the epiblast *in vivo *shortly after implantation. Despite the initially homogeneous state of the cells in the model and high Activin concentration during endodermal specification, there remains a cell subpopulation that does not reach the endodermal state. This simple model developed by us can be used to study the origins of cellular heterogeneity during germ layer specification.

## INTRODUCTION

Embryonic stem cells (ESCs), which were first derived more than 40 years ago, are remarkable in their ability to self-renew and differentiate into all types of somatic cells [1, 2]. The discovery of induced pluripotent stem cells (iPSCs) in 2006 was a real breakthrough in the stem cell field. iPSCs are similar to ESCs in most aspects, but they originate from differentiated somatic cells by being converted to the early pluripotent state by the exogenous expression of Oct4, Sox2, Klf4, and c-Myc. [3, 4]. Both ESCs and iPSCs correspond to the pluripotent epiblast before implantation [5, 6]. During mouse development, the epiblast emerges, along with primitive endoderm and trophectoderm on embryonic day 4.5 (E4.5) [7, 8]. After implantation, due to the alterations in their expression profiles, epiblast cells become receptive to external signals that prod them to proceed with differentiation into ecto-, meso-, and endoderm [9]. At E6.5, the gastrulation process mediated by FGF, Wnt, BMP, and Activin/Nodal signaling leads to the formation of the primitive streak in the posterior epiblast [10, 11, 12, 13, 14, 15, 16]. This structure, which is formed by cells undergoing the epithelial-to-mesenchymal transition, subsequently produces the mesoderm and definitive endoderm (DE) [17, 18]. DE is established in the distal part of the primitive streak, where Activin/ Nodal signaling, which is ensured by the visceral endoderm, shows the strongest effect and is more potent than the BMP signal produced by the extraembryonic ectoderm [17, 19]. Accordingly, applying high Activin doses should promote ESC differentiation into DE *in vitro *[20, 21]. The transcription factors Foxa2, Eomes, and Sox17 are responsible for DE formation [22, 23, 24, 25, 26, 27]. Interestingly, several reports have indicated a possible role for the core markers of ESCs – Oct4, Sox2, and Nanog – not only in the maintenance of the pluripotent state, but also in lineage specification [28, 29, 30, 31, 32]. It has been suggested that Nanog, which is also a target for Activin/Nodal signaling, can facilitate DE specification [33, 34, 35].

The future of regenerative medicine depends on ESCs and iPSCs; however, safe, efficient, and reproducible protocols for the *in vitro *differentiation of these cells must be developed before the cells can be used in practice. Several such protocols which mimic early embryogenesis are already available. First, culturing of ESCs/iPSCs in the chemically defined N2B27 medium allows one to dispose of undefined serum components; then, addition of the leukemia inhibitory factor (LIF), MEK inhibitor PD0325901, and GSK3 inhibitor CHIR99021 to this 2i-LIF-N2B27 medium promotes the propagation of the so-called “naïve” ESCs, which are homogeneous and have a transcription profile that corresponds to that in the pre-implantation epiblast at E4.5 [5, 36]. These cultivation conditions are usually applied in a limited experimental time, since prolonged culturing leads to epigenetic and genomic changes in ESCs [37, 38]. Subsequent replacement of this medium with the N2B27 medium, supplemented with bFGF, Activin, and knockout serum replacement (KSR), for two days promotes the transition of “naïve” ESCs to the “formative” pluripotent state, designated as the epiblast- like cells (EpiLCs). EpiLCs correspond to the epiblast of an implanted embryo at E5.5 and are capable of forming both primordial germ cells (PGCs) and derivatives of primary germ layers [6, 39, 40, 41, 42]. The chemically defined medium that facilitates the maintenance of a stable formative pluripotent state has been described in several recent publications [43, 44, 45, 46].

Here, we applied the Naïve-to-EpiLC transition protocol with addition of high doses of Activin to trigger DE specification. This strategy allowed us to derive DE precursors efficiently and reproducibly. Importantly, a homogeneous cell culture and the use of the Naïve-to-EpiLC transition scheme make this differentiation highly similar to that occurring *in vivo*. Additionally, we have derived a reporter ESC line that allows one to monitor the DE specification process in living cells. It would seem that the addition of a given growth factor should lead to that particular growth factor’s cellular specification. Thus, if we use a homogeneous 2D ESCs culture and add some of these signals, we can expect a homogeneous response and one-way specification. Yet, irrespective of the Activin concentration, we could not derive DE with 100% efficiency. The reaction–diffusion model in [47] might be able to help explain this.

## EXPERIMENTAL

**Plasmid construction**

The left and right homology arms (941 bp and 810 bp, respectively) near the stop codon of the* Foxa2 *gene were amplified from mouse genome DNA using Phusion DNA polymerase (ThermoFisher, USA). Next, the arms were ligated into the Oct4-TA2-EGFP vector (produced by A.A. Kuzmin, unpublished data) to replace the Oct4 locus-specific arms. The arms were ligated at the AvrII, NsiI, MluI, and SalI restriction sites. The guide-RNA sequences for the CRISPR-mediated DNA double-strand break were selected using the Benchling platform (benchling.com) in the region near the stop codon of the mouse *Foxa2 *gene. The selected guide RNA had the lowest probability of nonspecific activity, according to the method proposed by Hsu et al. [48]. The chosen guide was purchased from Evrogen (Russia), annealed, and ligated in the lentiCRISPRv2 vector (Addgene). All the final constructs were verified by Sanger sequencing. *Table 1* lists all the oligonucleotides used.

**Table 1:** Oligonucleotides used for CRISPR/Cas9

Name	Sequence
cM_LA-Foxa2_F	(AvrII) TATcctaggGACATACCGACGCAGCTACA
cM_LA-Foxa2_R	(NsiI) TATatgcatGGATGAGTTCATAATAGGCCTGGA
cM_RA-Foxa2_F	(MluI) TATcgcgttAGAGAAGATGGCTTTCAGGCCC
cM_RA-Foxa2_R	(SalI) ATAgtcgacTATTGACCCCGTCTCCCACA
Foxa2_guide_F	caccgATGAACTCATCCTAAGAAGA
Foxa2_guide_R	aaacTCTTCTTAGGATGAGTTCATc
gtM_FoxA2-F3	CAGTCACGAACAAAGCGGGC
gtM-FoxA2-R2	TCAGCGCATCTCCCAGTAAC

**Cell culture**

Unless specified otherwise, all cell culture products were purchased from ThermoFisher Scientific (Gibco, USA). Murine E14 Tg2a ESCs (Bay Genomics, USA) were grown at 37°C with 5% CO_2_. Cells were passaged using 0.05% Trypsin–0.01% EDTA solution under standard feeder-free conditions on gelatinized tissue culture dishes or plates in the mES medium: knockout Dulbecco’s modified Eagle’s medium (Knockout DMEM) supplemented with a 15% embryonic stem (ES) cell-qualified fetal bovine serum (Biosera, USA), 100 U/mL penicillin, 100 μg/mL streptomycin, 2 mM L-glutamine, 1× nonessential amino acids, 50 μM β-mercaptoethanol (Merck, Germany), and 500 U/mL of in-house bacterially expressed hLIF.

For the derivation of naïve ESCs, ESCs cultured in a serum-containing medium were seeded on poly-L-ornithine-treated (0.01%) plastic in the 2i-LIF-N2B27 medium: N2B27 supplemented with 500 U/mL hLIF, 3 µM CHIR99021 (Axon, USA), and 1 µM PD0325901 (Axon) as described in ref. [39]. For the derivation of EpiLCs, naïve ESCs were seeded on fibronectin (Merck)-coated (15 μg/mL) plastic in a EpiLC medium: N2B27 supplemented with 12 ng/mL bFGF (Peprotech, USA), 20 ng/mL Activin A (Peprotech), and 1% of the knockout serum replacement. For the RNA analysis, the cells were seeded at a density of 25,000 cells/cm². For the differentiation experiments, the cells were initially seeded at a low density of 250 cells/cm² in a EpiLC medium. After 2 days, the EpiLC medium was replaced with N2B27 with the addition of specific factors: 10 μM SB505124 (Tocris, UK) for ectoderm specification, 50 ng/mL BMP4 (Peprotech) for mesoderm specification, and 100 ng/mL of Activin A (Peprotech) for DE specification.

**Generation of the Foxa2::TA2-EGFP ESC line**

The Foxa2: :TA2-EGFP donor vector and lentiCRISPRv2 plasmid harboring gRNA (500 ng with a 1 : 1 molar ratio) were co-transfected in ESCs using a FuGENE transfection reagent (Promega, USA) in a OptiMEM medium. Next day, cells were transferred onto a 10 cm gelatinized dish. One day later, 2 μg/mL puromycin (Merck) was added to the culture medium for two additional days. The cells were cultured for an additional 10 days without the addition of selective antibiotics. Then, single clones were picked, expanded, and tested for transgene insertion by PCR using the gtM_FoxA2 primers and LR HS-PCR kit (Biolabmix, Russia). The EGFP level during the differentiation experiments was measured by flow cytometry on a CytoFLEX system (Beckman Coulter) and by time-lapse microscopy on a CQ1 confocal system (Yokogawa).

**Preparation of metaphase spreads**

The metaphase spread was prepared according to the previously described procedure [49]. Exponentially growing ESCs were treated with 0.1 µg/mL Colcemid (Sigma-Aldrich, USA) in a 5% CO_2_ incubator for 2 h at 37°C. Cells were collected and incubated in a hypotonic 0.56% KCl solution for 20 min, fixed in a methanol/acetic acid solution (3 : 1, v/v), washed, and stored in a fixative solution at −20°C. The cell suspension was dropped onto microscope glass slides (Superfrost; Thermo Scientific, Germany), air-dried, and kept overnight at room temperature in air. The metaphase spreads were then stained with DAPI and visualized on an EVOS fl Auto microscope.

**RNA isolation and RT-PCR**

Total RNA was isolated from the cells using the ExtractRNA reagent (Evrogen, Russia). For cDNA synthesis, 1 μg of total RNA was used. cDNA was synthesized using M-MuLV Reverse Transcriptase (Evrogen) and the oligo(dT) primer (Thermo Scientific). Quantitative RT-PCR was performed using a 5× qPCRmix-HS SYBR buffer (Evrogen) on a LightCycler 96 instrument (Roche, Switzerland). Expression levels were normalized to the endogenous GAPDH RNA level; dCq values were taken for visualization. The primers for RT-PCR are listed in *Table 2*.

**Table 2:** Oligonucleotides used for RT-PCR

Name	Sequence
Nanog_F	GCTCCATAACTTCGGGGAGG
Nanog_R	GTGCTAAAATGCGCATGGCT
Esrrb_F	GTCTGACACTTGGGGACCAG
Esrrb_R	CTACCAGGCGAGAGTGTTCC
Klf4_F	TACCCCTACACTGAGTCCCG
Klf4_R	GGAAAGGAGGGTAGTTGGGC
Fgf5_F	TCCTTCACCGTCACTGTTCC
Fgf5_R	TTCACTGGGCTGGGACTTCT
Otx2_F	ACTTGCCAGAATCCAGGGTG
Otx2_R	CTTCTTCTTGGCAGGCCTCA

**Immunocytochemistry**

Cells were fixed in 4% paraformaldehyde (ThermoFisher) for 10 min, permeabilized with 0.1% Triton X-100 for 15 min, blocked in 3% BSA for 1 h at room temperature, and stained with the appropriate antibodies (*Table 3*) overnight at 4°C. Samples were then washed five–six times with PBS plus 0.1% Tween (PBST), stained with secondary fluorescent antibodies (Jackson ImmunoResearch, USA) for 2 h at room temperature, and also washed with PBST. Immunostained cells were examined under an EVOS fl Auto fluorescent microscope (Life Technologies, ThermoFisher) equipped with DAPI, GFP, RFP, and CY5 filter cubes.

**Table 3:** Specific antibodies used in this study

Target	Cat. No	Manufacturer
Oct4	sc-5279 (C-10)	Santa-Cruz
Sox2	MA1-014	ThermoFisher
Nanog	A300-397	Bethyl
Foxa2	sc-374375	Santa-Cruz
Brachyury	AF2085	R&D Systems
Sox1	ab109290	Abcam
Sox17	AF1924	R&D Systems

## RESULTS

**Application of the Naïve-to-EpiLC transition of ESCs**

**Fig. 1:**
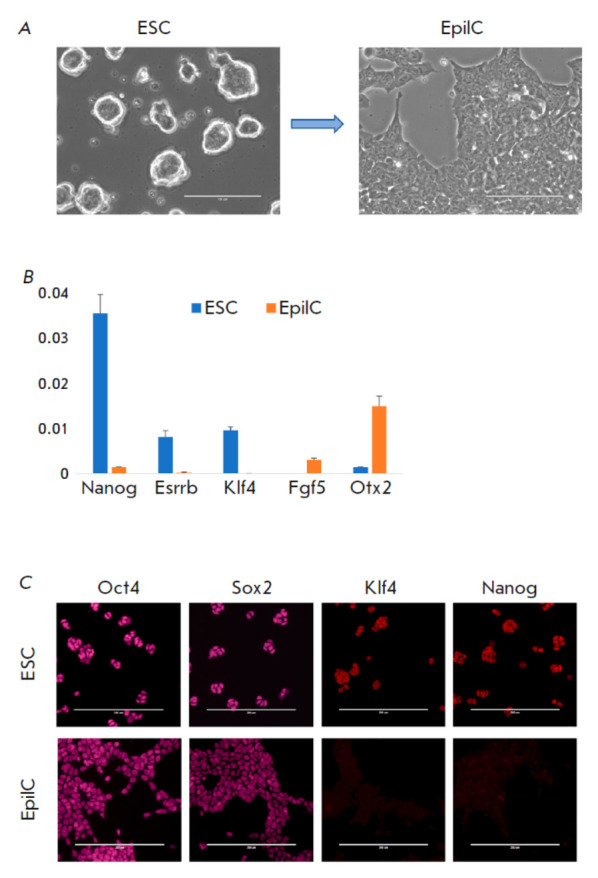
Monitoring the naїve-to-EpiLC transition of pluripotency *in vitro*. (*A*) Phase-contrast microphotography of ESCs and EpiLCs. Scale bar, 100 μm. (*B*) RT-PCR analysis of cells during the transition from naїve and primed markers of pluripotency. Delta Cq values are shown; the analysis was made in triplicates; the Gapdh RNA level served as a reference. (*C*) Results of the immunocytochemical analysis of the obtained cells with antibodies against Oct4, Sox2, Klf4, and Nanog. Scale bar, 200 μm

During cultivation in the defined 2i-LIF-N2B27 medium [36] for five days, ESC colonies were able to form round-shaped colonies without any signs of differentiated cells (*Fig. 1A*, left image). For the Naïveto- EpiLC transition, cells were seeded on the fibronectin- coated surface in the EpiLC medium for two days [39]. During this period, morphological changes were observed: cells became flattened and formed monolayer colonies (*Fig. 1A*, right image). The RNA and immunocytochemistry analysis confirmed the naïve and formative pluripotency states of these cells (*Fig. 1 B,C*). As expected, the naïve pluripotency markers Nanog, Esrrb, and Klf4 were expressed in ESCs but downregulated upon their differentiation to EpiLCs. Instead, the latter cells displayed the expression of primed pluripotency markers Fgf5 and Otx2 (*Fig. 1B*).

**Directing EpiLCs toward ectoderm, mesoderm, and endoderm**

**Fig. 2:**
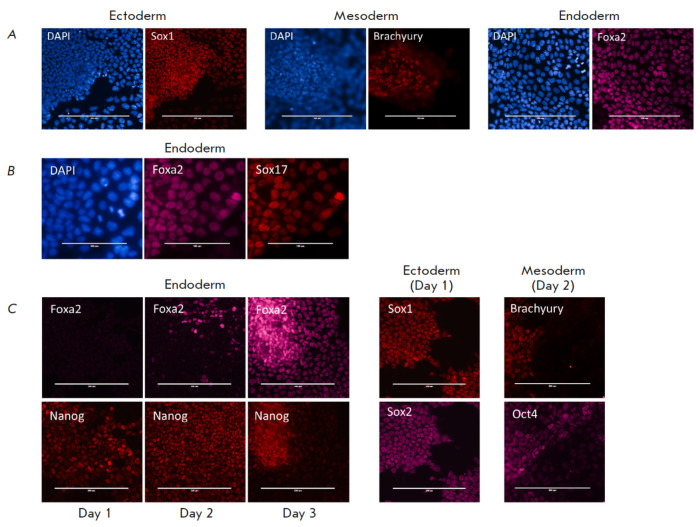
EpiLCs are receptive to external signals and can be directed toward three lineages. (*A*) Immunostaining of differentiated cells for Sox1, Brachyury, and Foxa2. Scale bar, 200 μm. (*B*) Immunostaining of endoderm derivatives for Foxa2 and Sox17. Scale bar, 100 μm. (*C*) Nanog re-expression during endoderm specification. Oct4 co-localizes with Brachyury during mesoderm specification, while Sox2 is co-stained with Sox1 during neuroectoderm differentiation. Scale bar, 200 μm

To direct EpiLCs toward distinct developmental trajectories, naïve ESCs were first seeded on the EpiLC medium at a low density. After two days, the medium was replaced with N2B27 supplemented with one of the following factors: the TGFβ-receptor inhibitor SB505124 (to promote ectoderm differentiation [34, 44, 50]), recombinant BMP4 (to promote mesoderm differentiation [51, 52]), or recombinant Activin A at a high concentration (100 ng/ml, to promote endoderm specification [20, 21, 43]). Immunostaining for lineage-specific markers revealed the successful onset of the desired differentiation trajectories: the mesendoderm marker Brachyury was detected in BMP4- treated cells; the neuroectoderm master-gene Sox1, in cells treated with SB inhibitor; and DE factor Foxa2, in Activin-treated cells (*Fig. 2A*). It appeared plausible that the Foxa2 could also mark cardiac progenitors, i.e. mesoderm lineage; hence, endoderm specification had to be additionally confirmed with Sox17 expression. The generated Foxa2+ cells indeed turned out to be positive for Sox17 (*Fig. 2B*).

During early embryogenesis, Nanog is downregulated at the implantation stage but is further re-expressed in the primitive streak region [53, 54, 55]. It has also been suggested that Nanog is needed for an appropriate DE differentiation through Eomes regulation [35]. Nanog expression, indeed, had disappeared in EpiLCs (*Fig. 1C*), reminiscent of its downregulation in the epiblast during implantation. To properly mimic the DE specification process *in vivo*, an *in vitro* model must feature Nanog re-expression. In our differentiation system, we observed Nanog re-expression as early as on Day 1 (*Fig. 2C*). At the same time, this expression preceded Foxa2 expression, which was detected on Day 2 and further increased by Day 3 of DE specification (*Fig. 2C*). While Oct4 and Sox2 function cooperatively in self-renewing ESCs, during the differentiation of these cells, the functions of the two factors diverge and are restricted to mesendoderm and neuroectoderm specification, respectively [30, 31]. Accordingly, we observed co-localization of Oct4 with the mesendoderm marker Brachyury and co-localization of Sox2 with the neuroectoderm marker Sox1 (*Fig. 2C*).

**Establishment of the reporter ESC line Foxa2::T2A-EGFP**

**Fig. 3:**
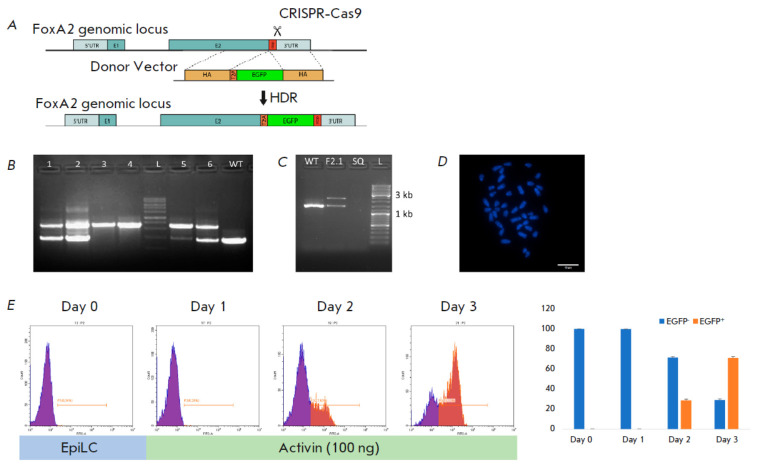
Establishment of the Foxa2::T2A-EGFP reporter ESC line. (*A*) Schematic representation of the targeting strategy. E1, E2 – exons; HA – homology arm; UTR – untranslated region; stop – stop codon. (*B*) Insertion verification by PCR in picked ESC clones. Genomic DNA of the parental cell line (WT) was used as a control. (*C*) Repeated insertion verification in the subcloned cell line. (*D*) Normal karyotype (40 XY) of the established reporter ESC line F2.1. Scale bar, 10 μm. (*E*) Left panel – flow cytometry analysis of EGFP expression of the F2.1 ESCs during DE specification; right panel – percentage of EGFP– and EGFP+ cells during DE differentiation; results are expressed as the mean of three replicates ± SD

For the purpose of live monitoring of DE specification, we inserted the T2A-EGFP cassette just in front of the stop codon within the last exon of the *Foxa2* gene using the CRISPR/Cas9-driven homology-directed repair (HDR) approach (*Fig. 3A*). This modification strategy has an obvious advantage over conventional gene targeting, which is rather inefficient [56, 57, 58]. In our case, CRISPR/Cas9 allowed accurate cassette insertion, producing chimeric Foxa2::T2A-EGFP mRNA. The presence of the T2A self-cleaving protein allows production of two distinct proteins (Foxa2 and EGFP), thus precluding the effects of EGFP on the Foxa2 functions. Furthermore, the Foxa2 and EGFP levels correlate, facilitating a rough quantification of the Foxa2 level by visualization of EGFP in living cells.

Following transfection with targeting plasmids, several ESC clones were chosen and verified for correct cassette insertion into the *Foxa2*locus (*Fig. 3B*). One of these clones (F2), the targeted one allele, was subcloned (F2.1, *Fig. 3C*). This subclone, showing a normal karyotype (*Fig. 3D*), was used in the subsequent experiments. We next performed EGFP visualization of F2.1 ESC differentiation into DE at different time points using flow cytometry analysis (*Fig. 3E*). Activation of EGFP was first observed on Day 2 of differentiation (29% of the cells), while the number of EGFP+ cells had increased to 71% by Day 3 of differentiation into DE (*Fig. 3E*, right panel). This result is consistent with the immunocytochemistry analysis of Foxa2 during DE specification (*Fig. 2C*).

**Heterogeneity induction in response to the single-growth factor**

**Fig. 4:**
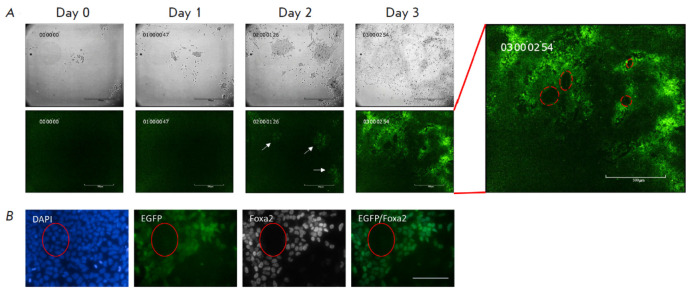
Live imaging of DE specification *in vitro*. (*A*) Time-lapse microscopy of the F2.1 ESCs during DE specification. Arrows indicate EGFP+ emerging cells. Scale bar, 500 μm. The time-lapse video can be found in the Supplementary material. (*B*) Co-localization of EGFP with Foxa2 following differentiation of the F2.1 ESCs into DE. Scale bar, 100 μm

Time-lapse microscopy was used to visualize DE specification in the living cells (*Fig. 4A*, Suppl. Video). In agreement with the results of the flow cytometry analysis, a EGFP signal was not observed within the first 24 h, implying some chromatin preparation for further specification. EGFP was detected for the first time 38 h after the addition of Activin to EpiLCs with the maximum amount of EGFP+ cells observed after 72 h of the treatment. The most interesting feature repeatedly noted throughout the experiments was the heterogeneity of the EGFP distribution across the cell population (*Fig. 4A*, right panel). The number of EGFP+ cells gradually increased during the specification and reached nearly 70%; however, it peaked at that level. Interestingly, the EGFP distribution did not show any bias towards the center or edge of colonies, as opposed to the previous studies where *in vitro* specifications as “micropatterns” was demonstrated [51, 59]. During immunocytochemical staining of the differentiated cell culture, we observed colocalization of the signal from antibodies with EGFP, proving the adequacy of the functioning of the resulting reporter cell line (*Fig. 4B*).

## DISCUSSION

Over the past decade, many valuable techniques of cultivating and differentiating pluripotent stem cells have been developed. These cells can now be maintained in various pluripotent states under chemically defined culture conditions and, importantly, precisely match the epiblast cells at different stages of embryonic development [5, 6, 44]. During recent years, several studies reporting *ex vivo *embryogenesis have appeared, including the establishment of blastoids, gastruloids, and even the whole embryos until embryonic day 8.5 (E8.5) [60, 61, 62, 63, 64, 65, 66, 67]. At the same time, modeling the simple and homogeneous processes of directed differentiation of pluripotent cells for the purpose of grasping the molecular mechanisms that underlie these processes, remains a worthwhile approach. Data obtained via this approach can then be extrapolated with a high probability of accuracy to embryonic development.

Here, we used chemically defined culture conditions to establish a simple and robust method for mouse ESC specification to DE. All the experiments were performed in a chemically defined serum-free medium (N2B27) purposely supplemented with various additional factors. We also established Foxa2::T2A-EGFP ESCs and demonstrated their usability during DE specification. We anticipate that the combination of chemically defined media and reporter cell lines will facilitate more comprehensive studies of the mechanisms that control lineage choice by pluripotent cells during the differentiation process.

New data challenging the paradigm that the transcription factors Oct4, Sox2, and Nanog act solely as guardians of the pluripotent state has recently appeared [30, 31, 32, 35]. Manipulations with the expression level of these factors in murine ESCs, indeed, without fail triggered differentiation toward extraembryonic tissues [68, 69, 70]; however, in human ESCs, these manipulations promoted a differentiation into primary germ layers [35, 71]. One can speculate that these differences are mostly related to naïve and primed pluripotent states rather than to species peculiarities. In this study, we have provided compelling evidence that Oct4, Sox2, and Nanog do not immediately disappear but transiently co-localize with known germ layer markers. Moreover, Nanog expression is re-activated during the DE specification. It would be of research value to modulate the level of this transcription factor during DE specification in future research, with the established F2.1 ESCs being a highly valuable tool in these attempts.

Differentiation to ectoderm, mesoderm, and definitive endoderm has been studied mostly in human ESCs, which are in the primed pluripotency state and correspond to the post-implantation epiblast [50, 52, 72, 73]. Meanwhile, murine ESCs are more complicated in this regard, as they are in the naïve pluripotency state and must be differentiated into the primed one prior to any specification of the germ layers. The situation has changed since the establishment of murine epiblast stem cells (EpiSCs), which correspond to epiblast cells after implantation and are similar to primed human ESCs [74, 75]. Subsequently, the ability of murine ESCs to transform into EpiSCs has been shown [39]. On their way to become EpiSCs, ESCs progress through the formative pluripotent state (EpiLCs), which corresponds to the epiblast right after implantation (E5.5) [41]. The most distinctive feature of EpiLCs resides in their ability to produce primordial germ cells (PGCs) [39, 76]. EpiLCs are homogeneous, and their expression profile makes them more suitable for embryogenesis modeling than EpiSCs. Besides, the latter cell type corresponds to the epiblast at E7.5, which is more committed [77]. Formative pluripotent stem cells have indeed been used for *in vitro *modeling of murine embryogenesis [40, 43, 44, 45, 51, 78] and can be regarded, in our view, as the golden standard in germ layer specification. Their homogeneous state also facilitates the precise deciphering of the mechanisms that underlie cellular specification. It is now obvious that this process is not controlled solely by the gradients of FGF, BMP, Wnt, and Nodal. Our study clearly shows that in excess of Activin and absence of any additional signals, EpiLCs do not uniformly reach the DE state. Hence, certain cell-autonomous stochastic processes also have to contribute to the specification of this lineage.

It seems to us that the Nodal–Lefty antagonism is not limited to the left-right asymmetry in mouse embryogenesis [79], but also operates in DE specification. It is known that Activin/Nodal signaling activates Lefty, which in turn inhibits this pathway, thereby ensuring the negative feedback mechanism [80]. This mechanism is a good example of the reaction– diffusion model [47], which explains the origin of heterogeneity in initially homogeneous systems [81]. According to this model, it would appear that there is about a 70% probability that adding Activin to EpiLCs would activate Nodal and just a 30% probability that it would activate Lefty. Further development of DE would proceed in accordance with the presence of an activator (Nodal) or an inhibitor (Lefty). Overall, the developed model could serve as a good starting point for further research into the mechanisms of heterogeneity onset during germ layer specification.

## CONCLUSIONS

The presented ESC – EpiLC – DE transition *in vitro* closely resembles DE maturation during embryogenesis. The transcription factor Nanog is downregulated in EpiLCs but is re-expressed in DE precursors. Despite the defined *in vitro *conditions of DE differentiation, only 70% of cells enter this developmental state. The molecular mechanisms underlying this phenomenon require clarification through future research.

Supplementary video. Time-lapse microscopy of DE specification *in vitro*. Registration was started at the timepoint when Activin A was added to EpiLC. Available at https://doi.org/10.32607/actanaturae.27510.

## Abbreviations

ESCs: embryonic stem cells
FGF: fibroblast growth factor
BMP: bone morphogenic protein
EGFP: enhanced green fluorescent protein
DE: definitive endoderm
iPSCs: induced pluripotent stem cells
LIF: leukemia inhibitory factor
EpiLCs: epiblast-like stem cells
EpiSCs: epiblast stem cells
PGCs: primordial germ cells
DNA: deoxyribonucleic acid
RNA: ribonucleic acid
KSR: knockout serum replacement
TGFβ: transforming growth factor beta
